# Re-discover student engagement from the perspective of definition and influencing factors

**DOI:** 10.3389/fpsyg.2024.1428668

**Published:** 2025-01-15

**Authors:** Qian Wang

**Affiliations:** Faculty of Information, Yunnan Normal University, Kunming, China

**Keywords:** student engagement, self-control, self-efficacy, learning activities, teacher-student relationship

## Abstract

In recent, the topic of student engagement has received a great deal of academic attention. However, there are numerous definitions of student engagement. Will this lead to inaccuracies and ambiguities in future definitions of student engagement? Therefore, it is important to have a common understanding of student engagement. In this paper, I present three definitions of student engagement that have the potential to be widely accepted. Additionally, in order to study student engagement in more depth, it is crucial to focus factors that influence student engagement. In this paper, 30 articles from three databases, Google Scholar, Taylor & Francis Online, and SAGE, were screened for data analysis based on the inclusion criteria. Three influences were extracted from the included articles, namely student self-control, teacher empathy, and learning environment, which were analyzed as possible indirect influences. An interesting finding is that the learning environment may act as a direct influence. Meanwhile, in order to improve student engagement, this paper draws on Schneider and Ingram’s categorization of policy tools, e.g., authority, incentive, and capacity tools, then formulates a causal model of the influences on student engagement, as well as provides a number of interventions, and finally offers some insights.

## 1 Introduction

There’s a part in the Battle of Hogwarts in Harry Potter where the teachers are organizing the students to reach the evacuation point,

*Ernie Macmillan stood up at the Hufflepuff table and shouted, “And what if we want to stay and fight?” There was a smattering of applause. “If you are of age, you may stay,” said Professor McGonagall*. (Rowling, 2007).

During the Battle of Hogwarts, those students who were actively involved in the battle began to see themselves as part of the school, from Ernie McMillan’s desire to fight in the battle to their peers responding with applause. These actions and thoughts are the perfect example of student engagement in the classroom. So what is the way or better way for teachers to get students as actively involved in classroom activities as Ernie McMillan was? One effective way may be for school leaders to encourage lecturers to experiment with new technological tools as an alternative to traditional teaching methods. In this way, students are likely to become actively engaged in the classroom.

Indeed, in the large body of research related to student engagement, scholars have interpreted the definition of engagement in different ways. For example, Kahu (2013) noted that “student engagement is viewed as an evolving construct that captures a range of institutional practices and student behaviors related to student satisfaction and achievement, including time on task, social and academic integration, and instructional practices” (p. 758). In his definition, Kahu focuses on student behaviors as well as instructional practices, possibly believing that behaviors have a greater impact on the effectiveness of student engagement, and therefore defines student engagement as an evolving structure that captures practices and behaviors. In another example, Sinatra et al. (2015) categorized student engagement into four dimensions “such as behavioral, cognitive, emotional, and agentic” (p. 2). Wong and Liem (2022) combined the above two examples of student engagement dimensions to categorize student engagement into learning engagement and school engagement, “learning engagement corresponds to the student’s work role, which represents the student’s active interaction with learning activities” (p. 118). While Fredricks et al. (2004) categorized student engagement into three categories:

Behavioral engagement is most commonly defined in three ways, [the first is]positive conduct, [the second is]involvement in learning and academic tasks, [and the third is]participation in school-related activities, research on cognitive engagement comes from the literature on school engagement and instruction, emotional engagement refers to students’ affective reactions in the classroom (Fredricks et al., 2004, pp. 62–63).

They classify student engagement as behavioral engagement, cognitive engagement, and emotional engagement (Fredricks et al., 2004). Again, this definition has been widely cited by others in academia (Wong and Liem, 2022; Fredricks et al., 2016; Kahu, 2013; Sinatra et al., 2015; Lin et al., 2024).

Of course, although Fredricks et al. (2004) categorized the definition of student engagement as being more in line with students’ cognitive developmental processes. However, Fredricks et al.’s (2004) definition seems to lack a focus on co-curricular engagement. For example, Fredricks et al. (2004) noted that “we include research on engagement in the classroom and in the larger school community” (p. 61). However, student engagement occurs not only in the classroom but also outside the school. In fact, other scholars have addressed this issue. For example, Engberg et al. (2016) mentioned that “Co-curricular engagement, however, shares a stronger relationship with the interpersonal dimensions of a global perspective” (p. 267). In other words, Engberg et al.'s (2016) argument indirectly suggested that the definition of student engagement should include off-campus. Similarly, Olewnik et al. (2023) considered the limitations of the place of definition of student engagement by adding off-campus engagement to the definition, “… co-curricular activities have been described in the literature and should be central to encouraging student participation” (p. 4).

Based on these considerations, how can student engagement be described more accurately and clearly? From this question, this paper attempts to describe a more compatible definition so that student engagement is not limited to the classroom. In addition, this paper categorizes and explains the influence factors of student engagement and suggests interventions. Given the current state of the field as briefly reviewed above, this article aims to answer the following questions: (1) What is student engagement? (2) What are the factors that influence student engagement? (3) What are the interventions to intervene in the influencing factors?

If we can answer the question, what is student engagement? Then we may be better able to assist teachers identify student engagement, which in turn may assist teachers assess student learning process. In addition, if we could develop learning strategies for different students, then we may be able to create a public resource library of learning strategies that help teachers and school leaders become more effective in their management.

The article is in three main sections. The first part of the article is the method, which screens the literature to be analyzed based on the inclusion and exclusion criteria. Subsequently, student engagement definitions as well as influencing factors are collected based on the screened literature, research hypotheses are developed, and each hypothesis is explained to develop a common understanding of student engagement definitions as well as influencing factors. Finally, the influencing factors were analyzed in depth to develop interventions and develop a causal model.

## 2 Method

### 2.1 Search strategy

Through access to Google Scholar, Taylor & Francis Online, and SAGE databases, an extensive literature search was conducted for research on student engagement. The search terms covered the three main categories of student engagement and were optimized by combining the use of Boolean operators so that the keywords I needed appeared in the articles. The final search terms were structured as “student engagement*,” “behavioral engagement,” “cognitive engagement,” and “emotional engagement.” Articles were limited to peer-reviewed English-language articles and were searched from January 2023 through December 2023. Through the search, the Taylor & Francis Online and SAGE databases did not have duplicate articles with the Google Scholar database. The initial search yielded approximately 1,070 articles.

### 2.2 Inclusion criteria

If the term empirical research appeared in the abstract, it was included in our review. This criterion ensured that the data were first-hand from the participants and allowed for a more visual examination of students’ performance in terms of behavioral, cognitive, and emotional engagement. On the other hand, if the abstract did not mention all three keywords, behavioral engagement, cognitive engagement, and emotional engagement at the same time, they were not included in the review. If all three keywords do not appear at the same time, the article may ignore the research on a particular keyword, thus biasing me against the research results.

Lastly, the participants of the study had to involve the student population. This criterion was included to ensure that the article findings were determined by direct observation of student behavior in the classroom as well as test scores to determine the level of student engagement, rather than being obtained through indirect descriptions by teachers or school leaders.

**Figure 1:**
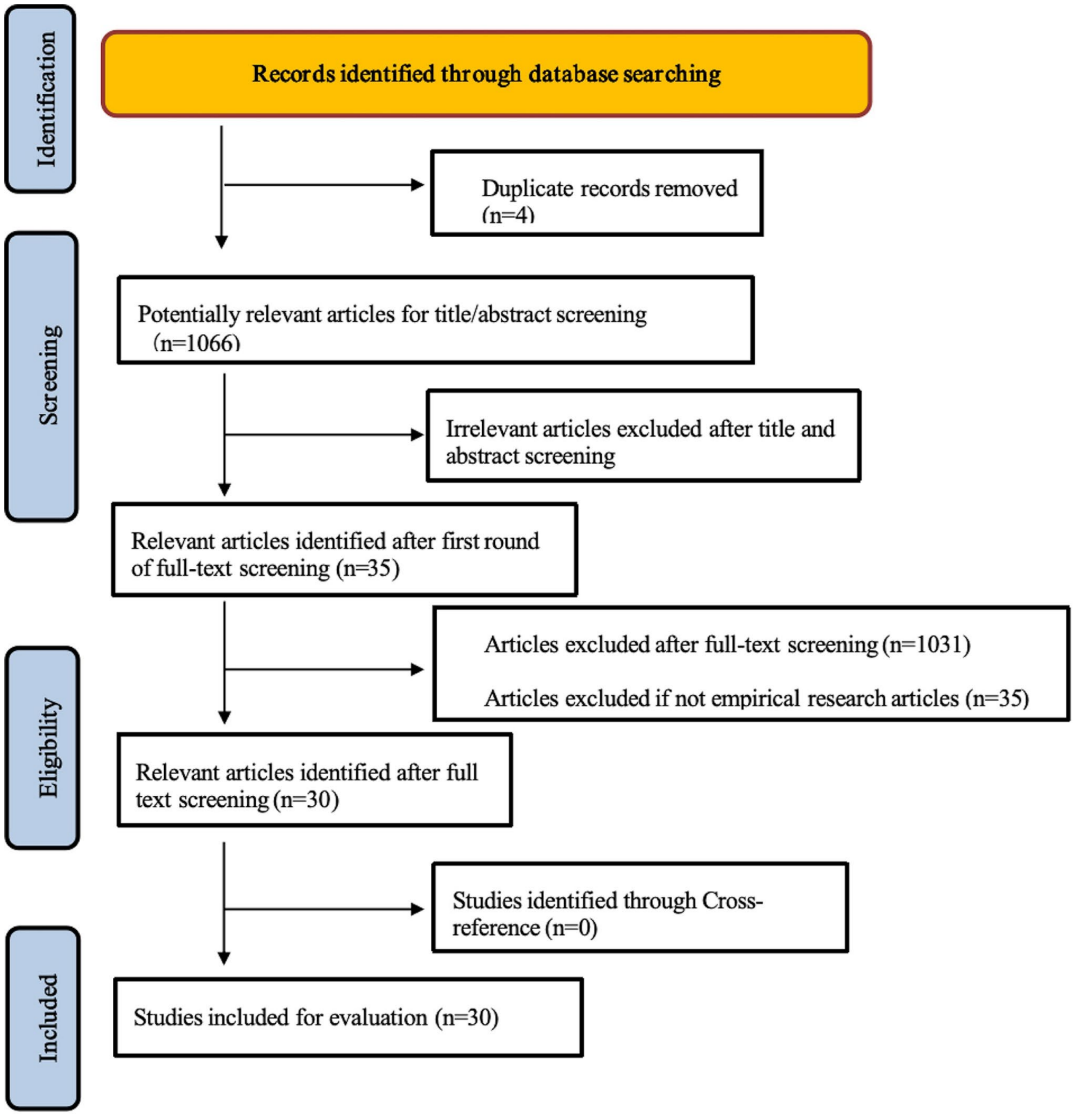
PRISMA flow diagram for article selection.

Exclude thesis, book chapters, preprints, editorials, and review articles, where the data is derived from an obvious experimental process to ensure that the article is empirical. Guided by the inclusion and exclusion criteria, I collected 30 existing studies. Each article was appropriately reviewed and validated, and the full text of the article was made available. Finally, to maximize transparency and traceability, I listed the basic structure and relevant evidence for all included articles (see Table 1). The article screening process follows the Preferred Project Report for Systematic Reviews and Meta-Analyses (PRISMA) declaration (Liberati, et al., 2009). Figure 1 illustrates the article selection process.

**Table 1:** Study characteristics of studies included in final sample.

ID	Article	Journal (or source)	Reasons for inclusion	Relevant evidence (In the spirit of honesty, we take direct quotes)
1	Salhab and Daher (2023)	European Journal of Investigation in Health, Psychology and Education	Research includes factors that influence student participation. The data comes from empirical research.	“Data from three focus group discussions and 15 semi-structured interviews…” (p.202).
2	Al-Obaydi et al. (2023)	Frontiers in Psychology	Research includes factors that influence student participation. The data comes from empirical research.	“The sample of the study consists of 114 EFL third-year college students.” (p. 1).
3	Su et al. (2023)	Journal of Computers in Education	Research includes factors that influence student participation. The data comes from empirical research.	“…to compare engagement among 147 students receiving blended learning and 137 receiving ERT at a local university…” (p. 445).
4	Thomas and Baral (2023)	The International Journal of Management Education	Research includes factors that influence student participation. The data comes from empirical research.	“…the behavioral, cognitive, and emotional pathways of learning engagement in a gamified management course through a within-subject experiment.” (p. 1).
5	Zhang et al. (2023)	Education and Information Technologies	Research includes factors that influence student participation. The data comes from empirical research.	“The quasi-experiment lasted for 6 weeks. The data from surveys, interviews, observations…” (p. 2091).
6	Hazzam and Wilkins (2023)	Computers and Education	Research includes factors that influence student participation. The data comes from empirical research.	“An online survey was used to obtain data from 659 higher education students in the United States…” (p. 1).
7	Vieites et al. (2023)	European Journal of Psychology of Education	Research includes factors that influence student participation. The data comes from empirical research.	“…impact of the MITCA method on school engagement in students in the 5th and 6th years of Primary Education” (p. 1283).
8	Tseng and Er (2024)	Educational Technology and Society	Research includes factors that influence student participation. The data comes from empirical research.	“Students in the experimental group (*n* = 26) performed the feedback practice with a regulated dialogic feedback approach…” (p. 133).
9	Halad and Veni (2023)	Bukittinggi International Conference on Education	Research includes factors that influence student participation. The data comes from empirical research.	“This study used an experimental quantitative research design with 32 junior high school students as its population.”(p.125).
10	Cabrera et al. (2023)	Learning and Individual Differences	Research includes factors that influence student participation. The data comes from empirical research.	“…by applying latent profile analysis (*N* = 1828) and student focus group interviews (*n* = 27).” (p. 1).
11	Li (2023)	Frontiers in Psychology	Research includes factors that influence student participation. The data comes from empirical research.	“A total of 413 Chinese EFL learners participated in the study and completed self-report measures…” (p. 1).
12	Mallik (2023)	Anatolian Journal of Education	Research includes factors that influence student participation. The data comes from empirical research.	“The data in the quantitative phase came from 157 undergraduate, graduate…” (p. 93).
13	Li et al. (2023)	Thinking Skills and Creativity	Research includes factors that influence student participation. The data comes from empirical research.	“A total of 714K-12 students in China’s Zhejiang Province participated in this study…” (p. 1).
14	Alzahrani (2023)	Journal of Language and Education	Research includes factors that influence student participation. The data comes from empirical research.	“Longitudinal self-report surveys (SRS) filled out by 127 undergraduate students after each class session throughout a four-week…” (p. 41).
15	Guo and Laokulrach (2023)	Nurture	Research includes factors that influence student participation. The data comes from empirical research.	“…data from the survey collected from 400 international undergraduate students in China and Thailand.” (p. 542).
16	Mann et al. (2023)	Frontiers in Psychology	Research includes factors that influence student participation. The data comes from empirical research.	“Year 9 was split into two cohorts who both participated in the study: one of which completed the Glengarry program in the first half of 2019…” (p. 1).
17	Sintya et al. (2023)	Proceeding Virtual English Education Students Conference	Research includes factors that influence student participation. The data comes from empirical research.	“The data were obtained through nonparticipant observation with the 11th grade in an EFL classroom at one of the senior high schools in Garut.” (p. 165).
18	Maina and Mberia (2023)	International Journal of Communication and Public Relation	Research includes factors that influence student participation. The data comes from empirical research.	“A sample of 384 students was drawn from the five public chartered universities’ main campuses, using a combination of various probability sampling techniques including stratified…” (p. 15).
19	Zhu et al. (2023)	Sustainability	Research includes factors that influence student participation. The data comes from empirical research.	“…by taking the Secondary School Geography Curriculum Standards and Textbooks Research, a small-scale private online course (SPOC) of the geography education…” (p. 1).
20	García-López et al. (2023)	Education Sciences	Research includes factors that influence student participation. The data comes from empirical research.	“This study compared the effects of gamification on engagement, cognition, metacognition, and…” (p. 1).
21	St-Amand et al. (2023)	Current Psychology	Research includes factors that influence student participation. The data comes from empirical research.	“The participants comprised 395 students (boys = 106; girls = 270; other = 8; NA = 11) (secondary school students = 291; primary school students = 97, NA = 7) from…” (p. 2499).
22	Wang et al. (2023)	The Scientific Temper	Research includes factors that influence student participation. The data comes from empirical research.	“Taking students from a university who participate in blended teaching of innovation and entrepreneurship as the research object…” (p. 570).
23	Bozan et al. (2023)	Technology, Knowledge and Learning	Research includes factors that influence student participation. The data comes from empirical research.	“Building on self-determination theory and self-system processes, we studied 329 student responses to a survey conducted…” (p. 509)
24	Yunita (2023)	Edumaspul – Jurnal Pendidikan	Research includes factors that influence student participation. The data comes from empirical research.	“The population is 84 students and the sample is 84 students with total sampling technique.” (p. 623).
25	Muallifah and Rohmatul (2023)	Prosiding The 6th National Conference of Genuine Psychology	Research includes factors that influence student participation. The data comes from empirical research.	“This study uses quantitative methods with sampling techniques; researchers use proportional random sampling techniques…” (p. 119).
26	Larasati (2023)	Al-Ishlah: Jurnal Pendidikan	Research includes factors that influence student participation. The data comes from empirical research.	“The study focuses on a sample of 36 students from the third social class.” (p. 3830).
27	Aliabadi and Weisi (2023)	International Journal of Educational Research Open	Research includes factors that influence student participation. The data comes from empirical research.	“Participants comprised 10 EFL teachers within the age range of 21–45 and the teaching experience of 1 to 16.” (p. 1).
28	Huang et al. (2023)	BMC Medical Education	Research includes factors that influence student participation. The data comes from empirical research.	“Methods We carried out a multi-center cross-sectional study among 10,901 medical students from 11 universities in China.” (p. 1).
29	Savitri et al. (2023)	Journal An-Nafs: Kajian Penelitian Psikologi	Research includes factors that influence student participation. The data comes from empirical research.	“The respondents of this research were 397 active university students in Bandung City.” (p. 249).
30	Fadilah et al. (2023)	Asian Journal of Research in Education and Social Sciences	Research includes factors that influence student participation. The data comes from empirical research.	“The data is collected using a quantitative method through the distribution of a questionnaire to QS degree students.” (p. 53).

## 3 Definition and description of student engagement

Some articles indicate a focus on the definition of student engagement through keywords in the abstract. For example, student engagement can be understood as the level of student engagement in the classroom (Al-Obaydi et al., 2023; Su et al., 2023; Cabrera et al., 2023), or a state of focus and engagement by students (Fredricks et al., 2004; Csikszentmihalyi and Nakamura, 2014). Regardless of whether these articles focus on the modality or the outcome of student engagement, the articles in the review reflect the following claims about the definition of student engagement:

1. Student engagement is the extent to which students have mastered their knowledge and the feedback given by the teacher to the students thus making them more engaged, the amount of energy that the students put into the task, and how the students connect the learning task.
2. Student engagement refers to the process of students joining in the learning task in a learning state that can be either conscious learning in the classroom or unconscious learning in life.
3. Student engagement contains behavioral engagement, cognitive engagement and, emotional engagement, and only by explaining the meaning of the three kinds of participation can the connotation of student participation be full.

Here, I will show a series of cited literature from different backgrounds to exemplify.

Regarding the first knowledge claim, Al-Obaydi et al. (2023) discuss the meaning of student engagement in terms of structured feedback between teachers and students. For example, Al-Obaydi et al. (2023) noted that “… the more students practice and get feedback from faculty and staff members on their writing and collaborative problem solving, the deeper they come to understand what they are learning”(p. 3). According to Al-Obaydi et al. (2023), the level of student engagement seems related to the structured feedback in order to motivate students to participate in the online classroom (Knowledge Claim 1). In terms of the level of student effort, the amount of energy put in by students is also part of engagement. Su et al. (2023) explained the definition of student engagement based on whether or not the student’s put in effort. For example, Su et al. (2023) wrote “Student engagement refers to the participation and energy that students devote to learning…” (p. 449). Su’s perspective emphasizes the energy that students devote to student engagement and focuses more on the level of student engagement (Knowledge Claim 1). Cabrera et al. (2023) further explained the relationship between student engagement and social psychology from a psychosocial perspective. For example, Cabrera et al. (2023) claimed that “student engagement in school is traditionally conceptualized as a socio-psychological construct that refers to how students connect to learning tasks” (p. 2). Here, Cabrera et al. (2023) understand student engagement as a connection to learning tasks, and how to make an effective connection becomes an indicator of the degree of commitment to student engagement (Knowledge Claim 1).

Regarding the second knowledge claim, student engagement is related to the state of flow in learning. Flow represents a state of complete inner absorption, a subjective state of total engagement (Fredricks et al., 2004; Csikszentmihalyi and Nakamura, 2014). Fredricks et al. (2004) noted that engagement in higher levels of activity represented a greater degree of engagement. However, this perspective makes the definition of student engagement limited. Because students are in greater level engagement when they enter a state of flow, whether they engage in a higher or lower level activity. In addition, Fredricks et al. (2004) argued that student engagement includes classroom engagement and school community engagement, which limits student engagement in classroom. However, student engagement occurs not only in the classroom, but also in off-campus or informal places (Carlson, 2023). Learning may occur unconsciously with student engagement. Therefore, the definition of student engagement could be understood as the process of completing a task in a learning state. This learning state could be a conscious learning state in the classroom or an unconscious learning state in life. Here, the learning state refers to students acquiring cognition, which could be knowledge in books or experiences in life. And the learning task could contain cognitive and skill-based learning tasks.

Regarding the third knowledge claim, it is necessary to explain the meaning of behavioral engagement, cognitive engagement, and emotional engagement separately. Fredricks et al. (2004) describe behavioral engagement as a positive expression of student engagement in learning and academic tasks as well as in school-related activities, whereas cognitive engagement is the student investment in learning as well as the students’ self-regulation strategies, in addition, emotional engagement refers to the students’ emotional responses in the classroom.

For behavioral engagement, some authors considered it as participating in learning activities and discussions, while others considered it as students’ physical participation in the target curriculum (Salhab and Daher, 2023; Al-Obaydi et al., 2023). Common to these interpretations is that behavioral engagement emphasizes observable behaviors and focuses on behaviors that are observable in the classroom. Combined with Fredricks’ view, behavioral engagement could be defined as the behaviors that students perform in response to the external environment with some cognition, which is observed and divided into conscious and unconscious behaviors. For cognitive engagement, first, the process of students setting goals and applying their thinking. Second, the improvement in students’ knowledge. Third, students’ effort and engagement in learning (Salhab and Daher, 2023; Al-Obaydi et al., 2023). The point is that these explanations are limited to describing the process by which students learn textbook knowledge and focus on the amount of effort students put to acquire knowledge and thus determine the level of cognitive engagement. In conjunction with Fredricks, cognitive engagement could be defined as the process by which students generate a state of flow, which is manifested in the form of both the integration of new cognitions into pre-existing cognitions and the alteration of pre-existing cognitions. For emotional engagement, some people believed that emotional engagement was the summative level of students’ emotions, while the other people believed that emotional engagement was the emotional development that students obtained by applying their knowledge and skills after learning the curriculum (Salhab and Daher, 2023; Su et al., 2023). Both of these explanations focus only on the students’ emotional state and ignore the process of emotion formation. Combined with Fredricks’ view, emotional engagement should be defined as the process of emotion formation.

To summarize, by reviewing articles I realized that the definition of student engagement is always confused by the word student. Because most researchers believe that students are those who sit at a desk and listen to a teacher, many definitions of student engagement are limited to the classroom. However, learning could happen anywhere and at any time. Therefore the role of the student could occur outside the school. The interpretation of student engagement should also include student engagement outside the school. Combined with the previous three definitions of student engagement, student engagement could be defined as the level of engagement in conscious and unconscious learning when students participate in on-campus and off-campus learning tasks. The level of engagement could be measured by behavioral engagement, cognitive engagement and emotional engagement.

Of course, I do not expect a broad consensus in the academic community, as there are too many conceptual definitions and controversies regarding the “what” of student engagement. If the authors had been more specific about where student engagement occurs, and if they had continued to analyze and integrate it, the definition of student engagement would have been more rigorously and consistently described. In order to gain a deeper understanding of student engagement, we need to explore what factors influence student engagement.

## 4 Influence factors of student engagement

There are many factors that influence student engagement. For example, Tseng and Er (2024), through their study of dialogic feedback, noted that students’ self-regulation skills are effective in transforming learning tasks, which affects the level of student engagement. In addition, Halad and Veni (2023) pointed out that social media flipped classrooms stimulate student activity and increase student engagement. Meanwhile, there are other authors who have studied the influences that include student attention, peer feedback, teacher-student interaction, and teaching mode (Salhab and Daher, 2023; Hazzam and Wilkins, 2023; Vieites et al., 2023).

Although many factors could be identified from the 30 articles, we wanted to focus on three factors: student self-control, teacher empathy, and learning environment. This choice was made for one main reason: in academia, fewer articles have examined the effects of student self-control, teacher empathy, and learning environment on student engagement (Wang et al., 2021). However, articles have suggested that all three factors have an impact on student engagement (Tangney et al., 2004; Xie and Derakhshan, 2021; Coller et al., 2011; Sinha et al., 2015). Without examining these three factors, we may miss opportunities to refine student engagement interventions. Therefore, the influencing factors we are looking at are the following three:

1. Student self-control influences student engagement by regulating emotions and performance.
2. Teacher empathy influences student engagement by facilitating the teacher-student relationship.
3. Learning environment influences student engagement through self-efficacy.

Here, I will show a series of examples by citing articles from a variety of backgrounds.

Student self-control has been defined as the ability to override or change one’s internal reactions, including impulses, emotions, thoughts, and behaviors (King and Gaerlan, 2013). King and Gaerlan (2013) demonstrated that self-control is associated with positive academic mood and negative academic mood. Prior to this, the association between self-control and emotion had not been fully explored, and self-control was more commonly studied in relation to academic performance. Building on this, Bertrams and Dickhauser (2009) also observed that “…individuals higher in self-control capacity are supposed to be better…in controlling their emotional expression…” (p. 136). This finding further describes the relationship between self-control and emotion. In addition to being related to emotions, self-control is also related to academic achievement. For example, Tangney et al. (2004) noted that students with high levels of self-control also have higher academic achievement, and that increased achievement positively contributes to student engagement in the classroom. Here, Tangney et al.’s (2004) argument suggests that self-control is not a direct influence but an indirect one. Similarly, this view is supported by Bai et al. (2022). For example, they suggest that high self-control motivates students to have better academic performance, which in turn promotes the level of student participation in the classroom.

Regarding the second knowledge statement, in terms of teacher empathy. Zhang (2022) defined empathy as an individual’s ability to appreciate and share the negative and positive emotions of others. Based on Zhang’s definition, Xie and Derakhshan (2021) showed that teacher empathy led to the formation of positive teacher-student interactions, which in turn led to the occurrence of student motivation and student engagement. Wang et al. (2021) found that teacher empathy engages students in learning, which leads to increased student engagement. Similarly, Hashim et al. (2014) argued that teachers’ use of empathy during classroom teacher process creates a positive interactive relationship between teachers and students, and students are able to feel cared for and understood by the teacher, which leads to more engagement in learning. The transfer of emotions is important in teacher-student interactions, and this transfer of emotions is often expressed in the teacher’s empathy. For example, Wang et al. (2022) stated that “language teaching and learning are both determined by an ocean of inner feelings, emotions, and internal psychological drives. This signifies the criticality of emotions in education and educational success” (p. 1). In short, teacher empathy is an indirect factor that affects student engagement by influencing the teacher-student relationship.

Finally, in terms of the learning environment. Learning environments are defined as external environments that influence learner learning (Shernoff and Vandell, 2007). Studies by Coller et al. (2011) and Sinha et al. (2015) demonstrated diverse learning environments, encompassing instructional game implementation scenarios, extracurricular art activities, and group work, which have been shown to have a facilitating effect on student engagement. For example, Shernoff and Vandell (2007) stated that “Teachers cannot control students’ engagement directly, but they may influence it indirectly by creating conditions in the learning environment facilitating it” (p. 4). Self-efficacy refers to a person’s beliefs about their ability to accomplish a task (Farmer et al., 2022). To explore the impact of the learning environment on student engagement even further, Sökmen (2019), in a study with information on the role of self-efficacy in the learning environment and student engagement, categorized the learning environment variables as shared control, student negotiation, promoting mutual respect, and teacher feedback. Student negotiation and promoting mutual respect variables influence student engagement through self-efficacy. In contrast, teacher feedback variables can directly influence student engagement. For example, Hyland (2003) noted that “teacher feedback…certainly it was used by most of the case study students in their immediate revisions to their drafts and was highly valued by all of them” (p. 228). Here, according to Sökmen (2019) and Hyland (2003), there is a new finding that learning environment can influence student engagement indirectly through self-efficiency as well as directly. Learning environment is an indirect variable as well as a direct variable.

In short, the learning environment can influence student engagement through self-efficacy when it is used as an indirect variable. On the contrary, when the learning environment serves as a direct variable, it can directly influence student engagement through itself. Thus, it is clear that the pathways through which the learning environment influences student engagement are more varied and complex. Based on the above research, how do we intervene in student engagement? Next, I will explore the policy categorization tool proposed by Schneider and Ingram (1990) and suggest some strategies for improving student engagement based on their categorization. Schneider and Ingram are discussed because their policy categorization tool is designed to move events in the expected direction of the policy text. This is consistent with my goal of proposing interventions.

## 5 Tools to improve student engagement

### 5.1 Schneider and Ingram’s classification of intervention tools

Based on the 30 pieces of literature I screened and my own life experiences, I summarized the intervention strategies that could improve student engagement. Based on Schneider and Ingram’s (1990) theory, I categorized intervention strategies into five categories: authority tools, incentive tools, capability tools, symbolic tools, and learning tools, and I categorized the target audience for the implementation of the tools into three levels: students, teachers, and schools. Corresponding to these five tool categories as well as the three levels, I categorized the 30 literature included into different intervention strategy tools as shown in Table 2.

**Table 2:** Tool measures.

Tool type	Schools	Teachers	Students
Authority tools	1. Develop educational strategies 2. Plan school development programs (Al-Obaydi et al., 2023; Aliabadi and Weisi, 2023; Huang et al., 2023; Fadilah et al., 2023)	Establish class rules and class motto	Null
Incentive tools	1. Encourage teachers to experiment with new technological tools 2. Encourage teachers to innovate teaching models and actively participate in teaching innovation and exploration (Salhab and Daher, 2023; Hazzam and Wilkins, 2023; Wang et al., 2023; Thomas and Baral, 2023; Zhang,2022; Halad and Veni, 2023; Cabrera et al., 2023; Li et al., 2023; Alzahrani, 2023; Mann et al., 2023; García-López et al., 2023).	Assign high quality homework and encourage students to complete it (Vieites et al., 2023; Li, 2023)	Develop a learning plan
Capacity tools	1. Provide specialized training for teachers, such as training for online instruction 2. General training programs for teachers 3. Self-awareness and self-esteem training as well as psychological counseling for university students (Hazzam and Wilkins, 2023; Mallik, 2023; Su et al., 2023; Savitri et al., 2023)	Develop teachers’ language skills, leadership skills, intercultural communication skills and sense of humor in skills training (Hazzam and Wilkins, 2023; Guo and Laokulrach, 2023; Sintya et al., 2023; St-Amand et al., 2023)	Null
Symbolic tools	1. Post slogans 2. Set up a university newspaper 3. Invest in modern information dissemination channels such as university websites, mobile apps and social media platforms (Maina and Mberia, 2023; Bozan et al., 2023; Yunita, 2023; Muallifah and Rohmatul, 2023)	1. Conduct class meetings on different topics 2. Set up class blackboard posters	Post quotes from famous people
Learning tools	Allow teachers to manage their classes according to the characteristics of their students (Larasati, 2023)	1. Allow students to use peer assessment for self-awareness 2. Allow students to engage in hands-on learning (Tseng and Er, 2024; Zhu et al., 2023)	Null

### 5.2 Causal modeling of intervention strategies

**Figure 2:**
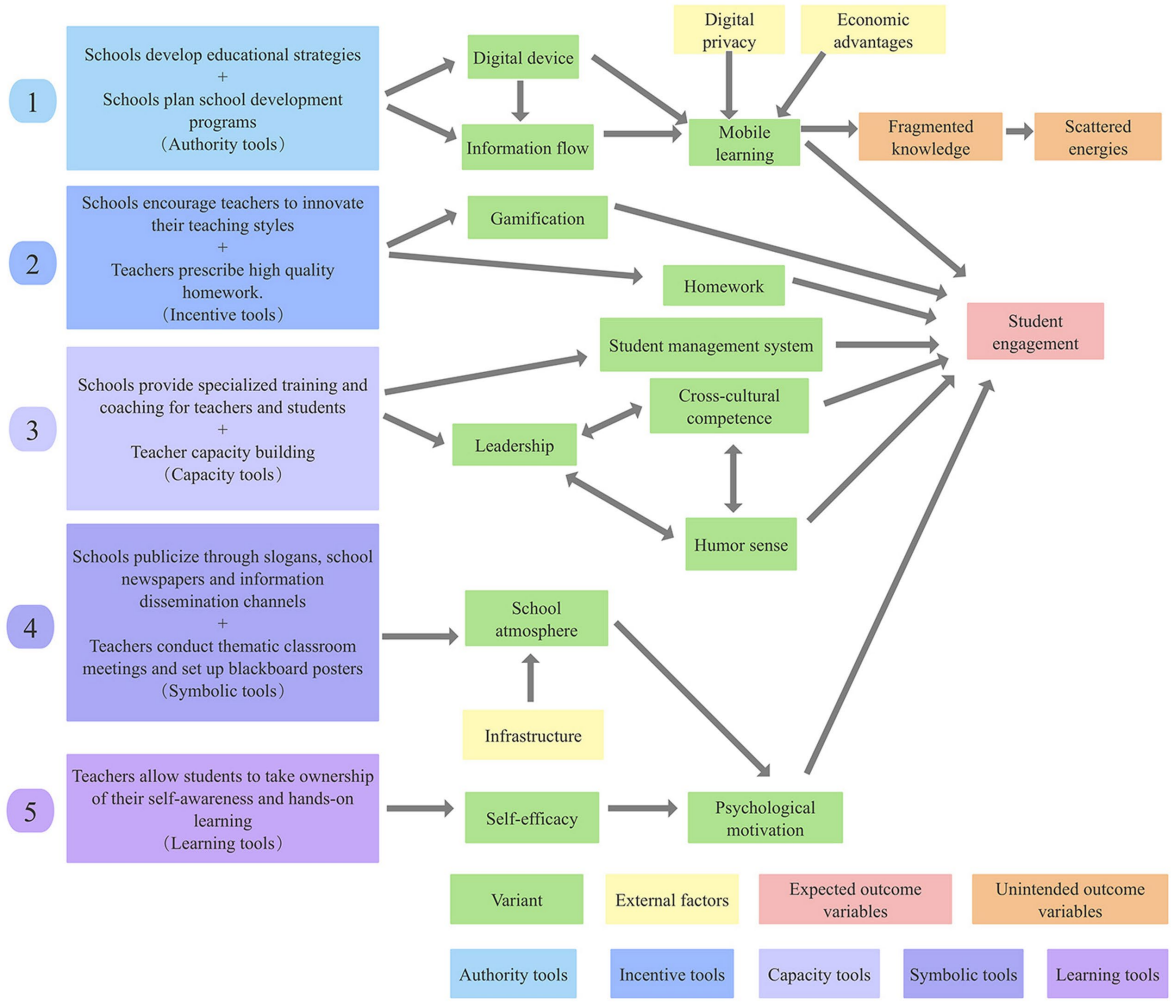
Causal model diagram. The number “1” in the model diagram represents the first part of the model diagram. The other numbers follow.

First, to identify the variables, I found some possible interventions and variables based on 30 articles (peer review) as well as personal experience (ideas). Second, I listed possible causal relationships between different variables. Finally, the links between variables were repeatedly confirmed and the final causal model diagram was developed by marking the instrument type, variables, external factors, expected outcome variables, and unintended outcome variables with different colors. This is shown in Figure 2. Next, I will explain how the variables in the causal model diagram affect student engagement.

In the first part of the model diagram, I viewed schools as authoritative tools for formulating educational policies and planning school development programs. This is in line with Schneider and Ingram’s (1990) definition. Policies set by the school affect the planning of digital devices in the classroom and the effectiveness of information flow between students, teachers, and the schools. As Maina and Mberia (2023) stated “Public universities should establish clear and comprehensive communication policies that outline the channels, frequency, and modes of communication between the institution, faculty, and students” (p. 25). If the information flow is not smooth, it will affect the effectiveness of students’ m-learning. In addition to this, external factors that affect the process of mobile learning experience are students’ digital privacy and economic advantages. According to Salhab and Daher (2023) “The current study results indicate that m-learning enriches students’ engagement” (p. 212). Mobile learning could influence the outcome variable of students’ engagement. However, the creation of m-learning may lead to the unintended outcome variable, i.e., the creation of fragmented knowledge, which leads to the distraction of students’ learning efforts (Su et al., 2023). The unintended outcome variable here refers to the possibility of other outcomes in addition to the outcome of enhanced student engagement.

In the second part of the model diagram, I consider the school’s encouragement of teachers to innovate their teaching models and teachers’ development of high-quality assignments as motivational tools. These two specific measures respond to Schneider and Ingram’s (1990) definition. According to Thomas and Baral (2023), “The findings divulge that gamified instructional design results in significantly higher levels of learning engagement than traditional and quiz-based instructional designs” (p. 9). Schools encourage teachers to be innovative in their instructional models, and teachers’ innovative use of gamified instruction affects student engagement. This is because gamified instruction makes the classroom fun and engages students’ attention. In addition to new teaching styles, improved quality of homework is an intervention that affects student engagement over time. For example, Vieites et al. (2023) stated that “…the MITCA method could specify and complement practices and educational strategies specifically aimed at promoting or maintaining school engagement…” (p. 1293).

In the third part of the model diagram, I consider the school’s provision of specialized training and coaching for teachers and students, as well as teacher competence development, as capability tools. Among them, leadership skills, intercultural communication skills, sense of humor, and student management system variables are manifestations of the competency tools. Schools provide teachers with specialized and generalist training, and teachers are actively involved in skills training activities that focus on developing leadership skills, intercultural communication skills, and a sense of humor (Hazzam and Wilkins, 2023; Guo and Laokulrach, 2023; St-Amand et al., 2023). Improved teacher competence could effectively control the pace of the classroom, enrich students’ cultural literacy, and enhance their level of interest in the classroom. In addition to the strengthening of human resources, the building of physical resources is also important. According to Larasati (2023) “In short, LMS is good enough to facilitate student engagement emotionally” (p. 3839), the construction of a learning management system in schools could enhance emotional engagement.

In the fourth part of the model diagram, I regarded the school’s publicity through slogans and school newspaper-level information dissemination channels and the teacher’s conducting thematic class meetings to set up blackboards as symbolic tools. These two symbolic tools are in line with Schneider and Ingram’s (1990) interpretation of them. School communication channels and classroom culture are the common ways that could influence the school atmosphere, however, the infrastructure of the school is an external factor that also could influence the school atmosphere. According to Muallifah and Rohmatul (2023) mentioned that “This shows that a school climate that is built positively and supports students in schools can influence the level of student involvement in participating in schools. The level of student involvement in participating in school activities” (p. 128). Similarly, Su et al. (2023) and Bozan et al. (2023) suggested that in a positive school climate, students change their psychological motivation and influence their level of involvement in school.

In the fifth part of the model diagram, I consider teachers allowing students to take ownership of self-awareness and hands-on learning as learning tools. This is because these two measures correspond to Schneider and Ingram’s (1990) description of learning tools. The fulfillment of self-efficacy in the classroom could lead to positive feedback, which could motivate students to participate in the classroom. For example, Yunita (2023) mentioned “This explains that the variable self-efficacy with student engagement has a positive correlation with a very strong correlation coefficient” (p. 628). In the same year, Bozan et al. (2023) explored the relationship between psychological needs and student engagement. This was an expansion of Yunita (2023) study because the fulfillment of self-efficacy is a type of psychological need fulfillment. Bozan et al. (2023) concluded that “Student engagement is determined by the degree to which students perceive that their psychological needs for autonomy, competence, and relatedness are met as prescribed.”(p. 509). Teachers delegate authority to students so that students have the opportunity to engage in self-awareness and hands-on learning to fully understand themselves. This could affect students’ sense of self-efficacy, which could change their psychological motivation and affect their level of engagement in the classroom.

In brief, combining the content of 30 literature articles as well as personal experience, I designed five interventions of different tool types. Using the implementation of intervention strategies as a starting point, investigated what variables in the influence pathway may affect student engagement during implementation and categorized the types of variables, including variables, external factors, intended outcome variables, and unintended outcome variables. An attempt is made to identify where different variables are located in the influence pathway and to construct causal relationships between variables.

## 6 Conclusion

The purpose of this paper was to determine the definition of student engagement through a literature review and to identify the factors that influence student engagement as well as interventions to increase student engagement. This choice was made because there are so many definitions of student engagement that it is easy for teachers or students to use student engagement to spread misinformation. Of course, the paper only presents one definition that may be understood, as I do not expect the academic community to reach a consensus on this. Based on the clarification of definitions, understanding the influences on student engagement can help us explore the antecedents and consequences of student engagement even further. Based on the 30 articles screened, I chose three influences, student self-control, teacher empathy, and learning environment, that have been less studied but have greater research value and potential to be analyzed. The results concluded that student self-control and teacher empathy can indirectly influence student engagement, while the learning environment could both directly and indirectly influence student engagement.

Additionally, to more effectively influence student engagement, I developed interventions based on Schneider and Ingram’s categorization of tools. Intervention strategies were developed in five different types of tools: authority tools, incentive tools, capacity tools, symbolic tools, and learning tools. At the same time, I designed a causal model to explain the pathways through which intervention strategies affect student engagement. One of this paper’s limitations is that I did not expand the search of the database and the number of influences was not chosen enough to form a system of influences. Future research can expand the research sample database, such as Scope database, and explore differences in student engagement across different regions and cultures.
